# Efficient Downregulation of *Alk4* in Skeletal Muscle After Systemic Treatment with Conjugated siRNAs in a Mouse Model for Duchenne Muscular Dystrophy

**DOI:** 10.1089/nat.2022.0021

**Published:** 2023-02-01

**Authors:** Sarah Engelbeen, Svetlana Pasteuning-Vuhman, Joke Boertje-van der Meulen, Rubina Parmar, Klaus Charisse, Laura Sepp-Lorenzino, Muthiah Manoharan, Annemieke Aartsma-Rus, Maaike van Putten

**Affiliations:** ^1^ Department of Human Genetics, Leiden University Medical Center, Leiden, the Netherlands.; ^2^ Alnalym Pharmaceuticals, Cambridge, Massachusetts, USA.

**Keywords:** delivery, dystrophin, pathology, *mdx* mouse

## Abstract

Downregulation of genes involved in the secondary pathology of Duchenne muscular dystrophy, for example, inflammation, fibrosis, and adiposis, is an interesting approach to ameliorate degeneration of muscle and replacement by fibrotic and adiposis tissue. Small interfering RNAs (siRNAs) are able to downregulate target genes, however, delivery of siRNAs to skeletal muscle still remains a challenge. We investigated delivery of fully chemically modified, cholesterol-conjugated siRNAs targeting *Alk4*, a nontherapeutic target that is expressed highly in muscle. We observed that a single intravenous or intraperitoneal (IP) injection of 10 mg/kg resulted in significant downregulation of *Alk4* mRNA expression in skeletal muscles in both wild-type and *mdx* mice. Treatment with multiple IP injections of 10 mg/kg led to an overall reduction of *Alk4* expression, reaching significance in tibialis anterior (39.7% ± 6.2%), diaphragm (32.7% ± 5.8%), and liver (41.3% ± 29.9%) in *mdx* mice. Doubling of the siRNA dose did not further increase mRNA silencing in muscles of *mdx* mice. The chemically modified conjugated siRNAs used in this study are very promising for delivery to both nondystrophic and dystrophic muscles and could have major implications for treatment of muscular dystrophy pathology.

## Introduction

Duchenne muscular dystrophy (DMD) is a progressive neuromuscular disorder. It is caused by nonsense or frameshift mutations in the *DMD* gene coding for the dystrophin protein. Dystrophin is essential for stabilizing muscle fibers during contractions. In the absence of dystrophin, muscles undergo continuous damage resulting in replacement of muscle fibers by fibrotic and adipose tissues. As a result of muscle loss, DMD patients lose ambulation and need assisted ventilation during the second decade of life and eventually die in the third or fourth decade of life [1].

Many efforts have been made to develop therapies to restore dystrophin in DMD patients [2,3]. These include gene therapy, read-through therapy, and exon skipping. Gene therapy uses adeno-associated viral (AAV) vectors to introduce micro-dystrophins, containing only complementary DNA (cDNA) encoding essential domains of the dystrophin protein, into muscle cells. Currently AAV vectors encoding three slightly different variations of micro-dystrophins are evaluated in clinical trials [4]. Initial results suggested that treatment with rAAVrh74.MHCK7.micro-dystrophin induces transgene expression and it was well tolerated with only mild adverse events [5]. Read-through therapy promotes suppression of premature stop codons that result from nonsense mutations in the dystrophin mRNA, allowing synthesis of intact dystrophin proteins. The read-through drug Ataluren has received conditional marketing authorization for DMD patients with nonsense mutations 2 years of age and older from the European Medicines Agency (EMA) and is applicable to 13% of the patients [3,6,7].

The exon skipping therapy utilizes antisense oligonucleotides (AONs) to correct the disrupted reading frame of dystrophin transcripts by hiding particular exons from the RNA splicing machinery so that a shorter but semifunctional dystrophin protein can be formed [3,7]. Four exon skip drugs (eteplirsen, viltolarsen, golodirsen and casimersen) have been approved by the Food and Drug Administration (FDA) and one (viltolarsen) by the Japanese Ministry of Health, Labor, and Welfare.

Although dystrophin restoration therapies are promising, their success depends on the availability of intact muscle tissue [3,8]. Once this is lost and replaced by adipose and fibrotic tissues, as the disease progresses, these therapies will be less effective. Stop codon read through and exon skipping therapies target dystrophin transcripts, which are only produced in skeletal muscle. The micro-dystrophin cDNAs are under muscle-specific promotors and therefore only transcribed in skeletal muscle and heart. However, even if the micro-dystrophin were ubiquitously expressed, this would not revert adipose and fibrotic tissues back into skeletal muscle [3].

Therapies targeting the secondary pathology, that is, by interference of transcripts encoding proteins involved in key pathological pathways like inflammation, fibrosis, and adiposis, could potentially maintain or improve muscle quality and delay disease progression [8,9]. As such, they could be used in combination with dystrophin restoration therapies or as stand-alone therapy. Moreover, these therapies would not only be applicable to all DMD patients, but also to other muscular dystrophies as the replacement of muscle tissue by fat and fibrosis occurs in most muscular dystrophies.

Small interfering RNAs (siRNAs) are 20–25 nucleotides long RNAs that can interfere with gene expression. siRNAs interact with the RNA-induced silencing complex (RISC) and binding of the antisense strand of the siRNA to the target mRNA results in the cleaving of the target mRNA by the RISC. This results in degradation of target mRNA, which prevents translation into the target protein [10,11]. However, delivery of siRNAs to specific tissues, including muscles, remains a challenge [12,13]. Unmodified siRNAs have a short half-life and are rapidly cleared from the body [10,14]. Chemical modifications of siRNAs and conjugations to the siRNA are needed to not only avoid degradation and clearance, but also to improve transport to target tissues [10,14].

Delivery of siRNAs to skeletal muscle has been previously assessed by myostatin-targeting siRNAs that were highly modified and cholesterol conjugated [11], or that were conjugated to docosanoic acid [15]. Myostatin is a negative regulator of muscle growth and has been an interesting target for treating muscular dystrophies. In both studies, treatment of wild-type mice with myostatin-targeting siRNA resulted in downregulation of myostatin levels, leading to a significant increase in muscle mass, size, and force. It remains however unknown whether delivery of siRNAs to dystrophic muscle is as sufficient, or hampered by the presence of fibrotic tissue.

In this study, we investigated the efficacy of chemically modified conjugated siRNAs in targeting muscle tissue and downregulating expression of *Alk4* transcript in *mdx*, a commonly used mouse model for DMD, and wild-type mice. Myostatin functions through interaction with type II receptors, ActRIIA or ActRIIB, which is followed by interaction with type I receptors ALK4 or ALK5. Since *Alk4* is expressed in skeletal muscles and is a key component of the myostatin signaling pathway, it is an appropriate target to assess delivery efficiency of this class of siRNAs to skeletal muscle. Targeting *Alk4* is however not expected to be therapeutic as we previously observed that downregulation of *Alk4* with AONs results in muscle atrophy in *mdx* mice [16]. In this study, we show that our *Alk4*-targeting siRNAs are well tolerated and can efficiently target muscle cells, including the diaphragm, and result in downregulation of the transcript, both after single dose and longitudinal treatment.

## Material and Methods

### siRNA design

**FIG. 1.**
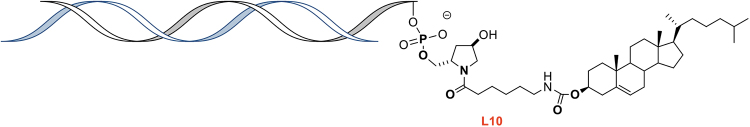
The sense (*dark gray*) and antisense (*light blue*) strands of the siRNAs are depicted. A L10 is conjugated to the 3′ end of the sense strand. L10, cholesterol ligand; siRNAs, small interfering RNAs.

Three siRNAs were designed to study delivery of siRNAs to skeletal muscles; two *Alk4*-targeting siRNAs (*Alk4* siRNA1 or *Alk4* siRNA2) and a control siRNA (targeting mouse/rat TTR) (Table 1). We included the TTR siRNA as a control for liver delivery to understand relative distribution of lipidated siRNAs to liver versus muscle. The sense and antisense strands had (E)-Vinylphosphonate [17], 2′Fluoro and 2′O-methyl modifications. At the 3′ end of the sense strand, cholesterol ligand is conjugated through an aminohexanoic acid spacer to a hydroxyproline scaffold [18]. The scaffold and the sense strand are connected using a phosphorothioate linkage (Fig. 1).

**Table 1.** siRNA Oligonucleotide Sequences and Chemistries Used

Duplex	Strand	Target	Oligonucleotide sequence	Transcript sequence
Control (m/rTTR)	S	mTTR	AfsasCfaGfuGfuUfCfUfuGfcUfcUfaUfaAfL10	AACAGUGUUCUUGCUCUAUAA
	AS	mTTR	VPusUfsaUfaGfaGfcAfagaAfcAfcUfgUfususu	UUAUAGAGCAAGAACACUGUUUU
siRNA1	S	ALK4	GfsasAfgAfuGfuGfAfAfgAfuUfuAfaGfcAfL10	GAAGAUGUGAAGAUUUAAGCA
	AS	ALK4	VPusGfscUfuAfaAfuCfuucAfcAfuCfuUfcscsu	UGCUUAAAUCUUCACAUCUUCCU
siRNA2	S	ALK4	gsasagauGfuGfAfAfgauuuaagcaL10	GAAGAUGUGAAGAUUUAAGCA
	AS	ALK4	VPusGfscuuAfaAfUfcuucAfcAfucuucscsu	UGCUUAAAUCUUCACAUCUUCCU

AS, antisense strand; L10, cholesterol ligand; *n,* 2′O-methyl nucleotide; Nf, 2′-fluoro nucleotide; s, phosphorothioate linkage; S, sense strand; siRNAs, small interfering RNAs; VP, (E)-vinylphopshonate.

### *In vitro* transfection and free uptake experiments

Mouse C2C12 myoblasts were plated on a 0.1 mg/mL PureCol collagen-coated (CellSystems Gmbh, Troisdorf, Germany) 6-well plate at a density of 200,000 cells/well in Dulbecco's modified Eagle's medium (DMEM) with 10% fetal bovine serum (FBS), 1% glucose, 2% Penicillin-Streptomycin (10,000 U/mL) and 2% GlutaMAX (Thermo Fisher Scientific, Waltham, MA) at 37°C with 10% CO_2_ in a humidified incubator. Cells were differentiated with differentiation medium containing DMEM, 2% FBS, 1% glucose, 2% Penicillin–Streptomycin (10,000 U/mL) and 2% GlutaMAX.

After differentiation into myotubes, delivery of either an siRNA targeting *Alk4* (*Alk4* siRNA1 or *Alk4* siRNA2 or a control siRNA (targeting mouse/rat TTR) was done in triplicate through transfection or free uptake.

Transfection was done at concentrations of 0.1, 1, and 10 nM in the following manner: siRNAs were mixed with lipofectamine 2000 (Thermo Fisher Scientific) and incubated at room temperature for 20 min. Afterward, the siRNA/lipofectamine mix was added to the cells and incubated for 3–4 h at 37°C. Cells were harvested with TriSure reagent (Bioline, London, UK) 24 h after transfection and stored at −80°C.

Gymnosis was done in myotubes at concentrations of 100, 300, and 1,000 nM of siRNA. CaCl_2_ was added to the differentiation medium to a final concentration of 9 mM to resemble physiological conditions. The siRNAs were mixed with the CaCl_2_-rich differentiation medium and added to the cells followed by incubation for 72 h at 37°C. Cells were then harvested with TriSure isolation reagent and stored at −80°C.

### Animals

Male *mdx* (C57BL/10ScSn-*Dmd^mdx^*/J) and wild-type (C57BL/6J) mice were bred at the animal facility of the Leiden University Medical Center. Mice were kept in individually ventilated cages with 12-h light/12-h dark cycles at 20.5°C and had *ad libitum* access to water and standard RM3 chow (SDS, Essex, United Kingdom). All experiments were approved by and performed following the guidelines of the Animal Experiment Committee of the Leiden University Medical Center.

### *In vivo* single dose siRNAs treatment

Mice were randomized over the different experimental groups (*n* = 3 *mdx* and wild-type males for each treatment group per mode of administration). Mice received either a single intravenous (IV) injection in the tail vein or intraperitoneal (IP) injection with 10 mg/kg of *Alk4* siRNA1, *Alk4* siRNA2, or control siRNA in 100 μL saline (0.9% NaCl) at the age of 5 weeks (*mdx*) and 3 months (wild type). One week after the injection, mice were sacrificed by cervical dislocation. Muscles (tibialis anterior, gastrocnemius, diaphragm, and heart) and liver were isolated and frozen in zirconium beads prefilled tubes (1.4 mm; OPS Diagnostics) in liquid nitrogen and stored at −80°C.

### *In vivo* systemic siRNA treatment

*mdx* males (*n* = 6 per group) received a weekly IP injection of 10 or 20 mg/kg of *Alk4* siRNA2 or control siRNA for the duration of 4 weeks starting at the age of 5 weeks. Wild-type males (*n* = 6 per group) received a weekly IP injection of 10 mg/kg of *Alk4* siRNA2 or control siRNA for the duration of 4 weeks starting at the age of 3 months. Four weeks after the final injection, blood samples were taken through a small cut in the tail vein and collected in Heparin-coated microvettes (Sarstedt, Nümbrecht, Germany). Plasma was obtained after centrifuging at 16,060 × *g* for 5 min at 4°C. The following markers for liver and kidney damage were measured using Reflotron strips and the Reflotron Sprint system (Roche Diagnostics); urea, glutamic oxaloacetic transaminase (GOT, also known as aspartate transaminase), glutamate pyruvate transaminase (GPT, also known as alanine aminotransferase), and alkaline phosphatase (ALP). Thereafter, mice were sacrificed by cervical dislocation. Tibialis anterior, gastrocnemius, diaphragm, heart, and liver were isolated, put in zirconium bead prefilled tubes (OPS Diagnostics LLC, Lebanon), frozen in liquid nitrogen and stored at −80°C.

### Gene expression analysis

The TriSure cell suspensions were thawed on ice and transferred to zirconium bead prefilled tubes (OPS Diagnostics) and homogenized with a MagNA lyser (Roche Diagnostics). Tissues were homogenized in the MagNA lyser in the presence of TriSure reagent (Bioline). Total RNA was isolated following the manufacturer's protocol. Subsequently, samples were purified using the NucleoSpin RNA Kit (Macherey-Nagel, Düren, Germany), according to the manufacturer's instructions. The cDNA was generated by incubation with random hexamer primers at 70°C, followed by an incubation at 42°C with dNTPs, BioScript reverse transcriptase and 5 × reaction buffer (Bioline).

Quantitative polymerase chain reaction (PCR) was performed in triplicate for each cDNA sample, in the presence of specific primers for *Alk4* and SensiMix SYBR (Bioline) with the LightCycler 480 system (Roche Diagnostics). Expression levels of *Alk4* were analyzed using the LinReg PCR software (version 2018.0) [19] and normalized for the expression levels of the housekeeping gene *Gapdh. Alk4* forward: CTGTTTGATTATCTGAACCG, *Alk4* reverse; AAGTCTCGATGAGCAATTCC, *Gapdh* forward; TCCATGACAACTTTGGCATTG, *Gapdh* reverse; TCACGCCACAGCTTTCCA.

### Statistics

Data analyses were performed with GraphPad Prism (GraphPad Software, San Diego, CA; version 8.1.1). All data are presented as mean ± standard deviation. A *P*-value of <0.05 was considered significant.

*Alk4* downregulation in C2C12 cells was assessed with a two-way analysis of variance (ANOVA) for the different concentrations and siRNAs. Variances between the different siRNAs for a certain concentration were analyzed with the Bonferroni's multiple comparison test. The *Alk4* levels in the different tissues after a single IV or IP injection were analyzed with a two-way ANOVA for the treatment method and the different siRNAs. Variances between the different siRNAs within a treatment method group were compared with the Bonferroni's multiple comparison test. Differences in *Alk4* expression after longitudinal administration were analyzed with a two-way ANOVA for the different tissues for the *mdx* groups. Expression levels between control and *Alk4* siRNA2 were analyzed with the Bonferroni's multiple comparison test. A Welch's *t*-test was used to analyze the differences in *Alk4* expression after longitudinal administration for the different tissues in the wild-type groups.

Markers for liver and kidney function were analyzed with a two-way ANOVA to compare the treatment dose and treatment groups for the *mdx* groups. Differences between the different siRNAs within treatment dose groups were compared with the Bonferroni's multiple comparison test. Levels of liver and kidney markers were compared with a Welch's *t*-test for the wild-type groups.

## Results

### siRNAs mediated downregulation of *Alk4* in myotubes

**FIG. 2.**
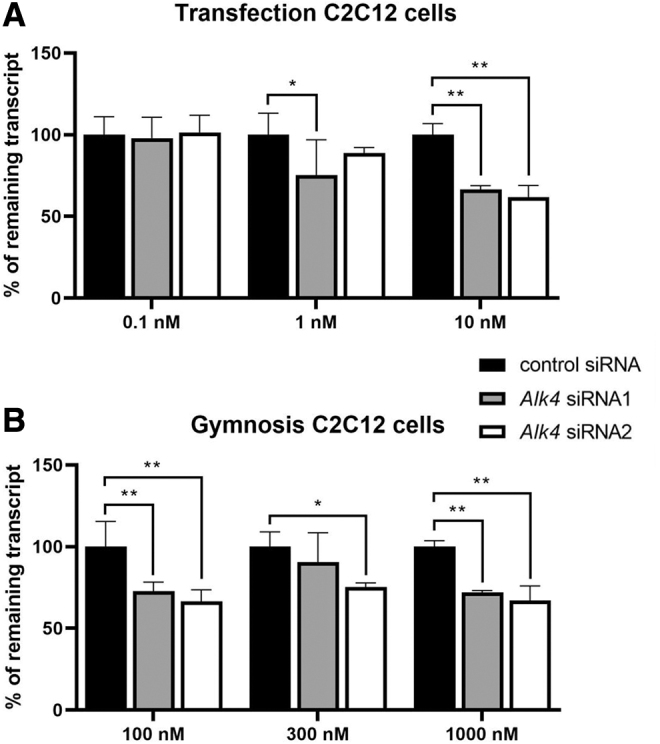
*Alk4* downregulation was assessed after **(A)** transfection and **(B)** gymnosis of C2C12 myotubes with *Alk4*-targeting siRNA1 and siRNA2 and a control siRNA. Analysis was performed with a two-way ANOVA comparing treatment dose and siRNA treatment followed by Bonferroni's multiple comparisons. *P < 0.05, **P < 0.01. ANOVA, analysis of variance.

To assess the applicability of chemically modified conjugated siRNAs to target muscle fibers, we transfected them in triplicate into myotubes at concentrations of 0.1, 1, and 10 nM or used gymnotic delivery at concentrations of 100, 300, and 1,000 nM. For *Alk4* siRNA experiments, control siRNA levels were used as a reference. Both *Alk4* siRNAs induced comparable but variable levels of downregulation for the different transfection doses. A significant decrease was observed at 1 nM of *Alk4* siRNA1 and at 10 nM for both *Alk4* siRNAs (Fig. 2A). In the gymnosis experiment, *Alk4* siRNA2 resulted in significant downregulation of *Alk4* regardless of the dose, whereas for *Alk4* siRNA1 this was achieved at 100 and 1,000 nM (Fig. 2B).

### Comparable levels of *Alk4* downregulation after a single IV or IP injection in *mdx* and wild-type mice

**FIG. 3.**
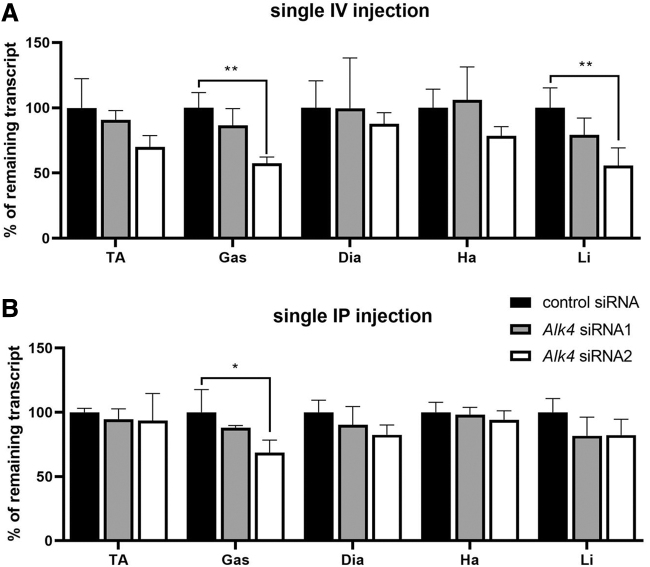
Systemic treatment of *Alk4* siRNAs in *mdx* mice (*n* = 3 per group). **(A)** *mdx* mice were treated with a single IV injection of 10 mg/kg siRNA. Remaining *Alk4* transcript was assessed by qPCR in the tibialis anterior (TA), gastrocnemius (Gas), diaphragm (Dia), heart (Ha), and liver (Li). **(B)** Remaining *Alk4* transcript after single IP injection of 10 mg/kg siRNA. A two-way ANOVA was performed to compare between route of administration and the different siRNAs, followed by the Bonferroni's multiple comparisons to compare between different groups. *P < 0.05, **P < 0.01. IP, intraperitoneal; IV, intravenous; qPCR, quantitative polymerase chain reaction.

To study the efficiency of chemically modified conjugated siRNAs to downregulate *Alk4* expression in tissues, we tested them *in vivo*. The *mdx* mice received a single dose of 10 mg/kg *Alk4* siRNA1, siRNA2, or control siRNA through IV or IP injection, at the age of 5 weeks. The *Alk4* siRNA1 did not have a noticeable effect on *Alk4* expression compared with the control siRNA after a single IV injection (Fig. 3A) or IP injection (Fig. 3B). In contrast, *Alk4* siRNA2 resulted in significant downregulation of *Alk4* of 42.5% ± 4.8% and 31.5% ± 9.7% in the gastrocnemius upon IV and IP administration, respectively, and of 44.3% ± 13.7% in the liver after IV injection. The liver was included as positive control since *Alk4* is highly expressed there.

The efficiency of the siRNAs to downregulate *Alk4* expression in nonfibrotic tissue was assessed in wild-type mice at the age of 3 months. Wild-type mice received a single dose of 10 mg/kg of *Alk4* siRNA1, siRNA2, or control siRNA through IV or IP injection. After a single IV injection, *Alk4* downregulation was most pronounced in *Alk4* siRNA2-treated mice, reaching significance in the heart (20.6% ± 8.1%) (Supplementary Fig. S1A). Contrastingly, single IP delivery of *Alk4* siRNA1 and siRNA2 more efficiently reduced *Alk4* transcript levels compared with single IV injection, reaching significance for the diaphragm (42.3% ± 12.3% siRNA1 and 43.3% ± 31.5% siRNA2), the gastrocnemius (35.7% ± 28.9%, siRNA2), and heart (18.2% ± 9.9%, siRNA2) (Supplementary Fig. S1B).

Given that siRNA2 was more effective in downregulating *Alk4* expression, and that IP delivery was more efficient in wild-type mice, we decided to continue with siRNA2 and IP injections.

### Efficient *Alk4* downregulation upon longitudinal systemic delivery

**FIG. 4.**
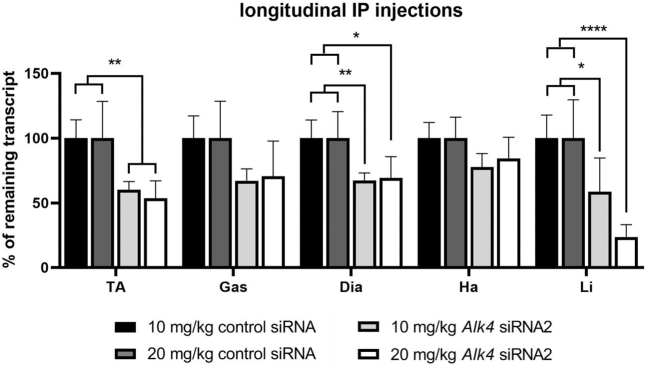
*Alk4* downregulation after longitudinal treatment with 10 or 20 mg/kg siRNA in *mdx* mice (*n* = 6 per group) resulted in significant downregulation in the tibialis anterior (TA), diaphragm (Dia), and liver (Li). In the gastrocnemius (Gas) and heart (Ha) a small and nonsignificant decrease in *Alk4* levels was observed. A two-way ANOVA was done comparing the treatment dose and siRNA treatment, followed by the Bonferroni's multiple comparison test. *P < 0.05, **P < 0.01, ****P < 0.0001.

To determine how effective siRNA2 is at downregulating *Alk4* mRNA expression after longitudinal treatment, we injected mice once weekly over a period of 4 weeks. Treatment was initiated at 5 weeks of age in *mdx* mice and at 3 months of age in wild-type mice. After systemic treatment of 10 mg/kg *Alk4* siRNA2, efficient downregulation of *Alk4* mRNA was observed in all tissues, compared with control siRNA. This reached significance for the tibialis anterior (39.7% ± 6.2%), diaphragm (32.7% ± 5.8%), and liver (41.3% ± 29.9%) in *mdx* mice (Fig. 4). Treatment with a higher dose of 20 mg/kg *Alk4* siRNA2 did not result in more efficient downregulation in muscle in *mdx* mice (Fig. 4). In wild-type mice, *Alk4* was significantly downregulated in all muscles and the liver upon treatment with 10 mg/kg *Alk4* siRNA2 (Supplementary Fig. S2).

**FIG. 5.**
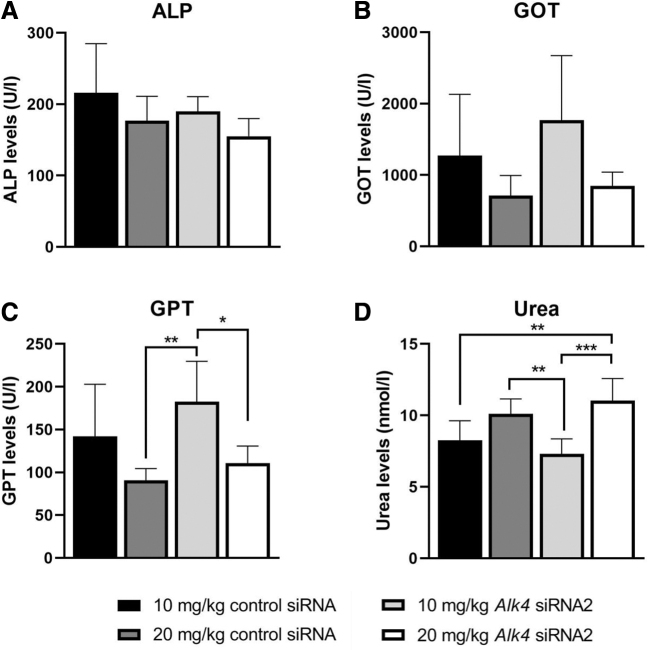
The tolerability of *Alk4*-targeting siRNA treatment was assessed by quantification of markers for kidney and liver function in *mdx* mice (*n* = 6 per group). A two-way ANOVA was done comparing the treatment dose and siRNA treatment, followed by the Bonferroni's multiple comparison test. *P < 0.05, **P < 0.01, ***P < 0.001. ALP, alkaline phosphatase; GOT, glutamic oxaloacetic transaminase; GPT, glutamic pyruvic transaminase.

We also assessed if long-term siRNA treatment is well tolerated by measuring several markers for liver and kidney function in plasma (Fig. 5 and Supplementary Fig. S3). Notably, phosphates and transaminases that are often used as liver damage markers, are also abundant in skeletal muscle and serum levels are elevated in DMD patients and *mdx* mice. However, treatment with *Alk4* siRNAs did not further increase levels of ALP and GOT markers in *mdx* mice (Fig. 5A, B). The levels of GPT were lowered after 20 mg/kg of control siRNA or *Alk4* siRNA compared with 10 mg/kg of *Alk4* siRNA (Fig. 5C). The highest dose of 20 mg/kg siRNA led to an increase in urea levels compared with 10 mg/kg of siRNA, regardless of the type of siRNA (Fig. 5D). No differences were observed in these markers in the plasma of wild-type mice treated with control siRNA and *Alk4* siRNA2 (Supplementary Fig. S3).

## Discussion

Restoration of dystrophin expression is considered the most promising approach to treat DMD. The availability of intact muscle tissue is however vital for the success of these therapies. Therefore, treating the secondary pathology to delay muscle deterioration and disease progression could be an interesting opportunity for an add-on therapy. Downregulation of specific target genes involved in fibrosis, inflammation, or adiposis could be achieved with siRNAs and AONs.

Protein knockdown can be achieved in different ways using either single-stranded AONs or double-stranded siRNAs. AONs can induce RNase H-mediated mRNA degradation or inhibit translation of mRNA into protein, while siRNAs act through the RISC resulting in cleavage of the target mRNA resulting in degradation [12,20]. Many efforts are being made to improve the delivery of AONs and siRNAs to target tissues. Chemical modifications to the sugars and backbone of the AON or guide strand of the siRNA can improve the metabolic stability, duration of action, potency, and target specificity. Alternatively, AONs or the guide strand of the siRNA can be conjugated with trivalent N-acetylgalactosamine (GalNAc), fatty acids, or cell-penetrating peptides [12]. While both modalities allow targeted reduction of protein production, which one is the best choice—or whether both are equally valid candidates—will vary based on the target transcript, target tissue, and site of action (cell nucleus vs. cytoplasm).

This study assessed the efficiency of new modified and conjugated-siRNAs to target skeletal muscles upon systemic administration. The therapeutic potential of siRNAs to downregulate gene expression has been confirmed in clinical trials for a variety of genes and diseases, leading to approval of five siRNAs (patisiran, givosiran, lumasiran, inclisiran and vultrisiran), whereas several other siRNA candidates are currently in Phase 3 clinical trials [21]. The currently approved siRNAs all target hepatocytes. To ensure delivery of these siRNAs to liver, either lipid nanoparticles (patisiran) or GalNAc conjugation (givosiran, lumasiran, inclisiran and vutrisiran) are used as a delivery platform. Lipid nanoparticles encapsulate the siRNAs protecting them from degradation. Due to their large size, they mainly end up in fenestrated tissues like the liver [22]. GalNAc binds with high specificity and affinity to the asialoglycoprotein receptor on hepatocytes making it an interesting approach to silence liver-specific genes [23]. However, siRNA delivery to tissues other than liver still remains a challenge [13,21,24,25].

Once the hurdles of delivery of siRNAs to skeletal muscle have been overcome, they could have great potential for the treatment of a large range of muscle diseases, including DMD. In the last decade, several developments have improved siRNA uptake by muscle tissue. Proof of principle to downregulate gene expression in skeletal muscle was obtained in studies using electropulsation to locally deliver siRNAs [26,27], and after IV injection of a limb that was temporarily isolated by a blood pressure cuff [28]. Stability of siRNAs against thermal and nuclease degradation has been improved by chemically modifying the siRNA backbones, sugars, and nucleobases [13]. Local delivery of a nanoparticle complex containing myostatin siRNAs and atelocollagen-induced myostatin downregulation in the masseter [29]. However, local delivery of siRNAs to skeletal muscle for a disease such as DMD is unfeasible since we have over 700 skeletal muscles and most are affected in DMD.

Two recent studies have investigated myostatin downregulation in muscle after systemic treatment with cholesterol-conjugated or docosanoic acid-conjugated siRNAs [11,15]. A single IV injection of 50 mg/kg of modified cholesterol-conjugated myostatin-targeting siRNAs resulted in 85%–90% reduction of myostatin expression in the gastrocnemius, triceps, and extensor digitorum longus up to 21 days postinjection in CD-1 wild-type mice [11]. Two subcutaneous injections of 20 mg/kg of docosanoic-conjugated myostatin-targeting siRNAs resulted in 38% downregulation of myostatin in the gastrocnemius, 43% in the triceps, and 65% in the heart up to 1 month post-treatment in FVB/NJ wild-type mice [15]. While both lipids achieve muscle delivery, their relative efficiency is difficult to assess as different sequences, chemical modifications, and chemical motifs were employed in these studies.

Conjugates to improve delivery to skeletal muscle are also under preclinical investigation for single-stranded splice modulating AONs to induce DMD exon skipping or to reduce toxic RNA aggregates in myotonic dystrophy. In this study, either monoclonal antibodies or fragment antibodies targeting transferrin are conjugated to the oligonucleotides (oral presentations from Dyne Therapeutics and Avidity BioScience at the annual Parent Project Muscular Dystrophy conference, June 2022).

Although these studies are very promising, they were only conducted in wild-type animals, and it remains unclear whether efficient siRNA delivery and gene downregulation is also feasible in a setting of dystrophic muscle. It is possible that delivery is inhibited due to deposition of fibrosis or reduced expression of muscle markers that are required for targeted delivery. However, in a study by Heemskerk *et al.*, it has been shown that AON levels after treatment are higher in dystrophic *mdx* muscles than in wild-type muscles. It is believed that this is the result of the continuous degeneration and regeneration cycles that make muscles more permeable [30]. In our study, we included both *mdx* and wild-type mice to evaluate whether our siRNAs could be efficiently delivered into dystrophic and nondystrophic muscle. We observed efficient *Alk4* downregulation after a single dose of *Alk4* siRNA–cholesterol conjugate, especially through the IP administration method. Furthermore, our results confirm that siRNAs can be delivered to dystrophic muscle efficiently using the lipid conjugation strategy.

It is especially encouraging that high knockdown was achieved after longitudinal treatment in the diaphragm, which is the most severely affected muscle in the *mdx* model. We cannot make a direct comparison between the results in *mdx* and wild-type mice since the experiments were performed at different ages. However, the fact that we observe greater *Alk4* downregulation in wild-type mice than in *mdx* mice suggests active rather than passive uptake of the siRNA conjugate. This is in contrast to unconjugated ASOs, which are taken up with higher efficiency by mdx mice due to the dystrophic pathology that makes muscles more permeable [30].

To investigate the additive effect of multiple dosing and increment of the dose, *mdx* mice received weekly IP injections of 10 or 20 mg/kg *Alk4* siRNA2 for a period of 4 weeks. Downregulation of *Alk4* was observed in all muscles, including the heart, reaching significance in the tibialis anterior, diaphragm, and liver. Whereas a single IP injection resulted in significant downregulation of *Alk4* in the gastrocnemius, there was only a limited amount of, and nonsignificant, *Alk4* downregulation in other skeletal muscles and liver. However, multiple dosing led to a more pronounced *Alk4* downregulation in all skeletal muscles. It has been previously reported that high level of Alk4 knockdown can induce skeletal muscle atrophy [16]. We did not observe this in our current study when comparing the muscle weights of the tibialis anterior and gastrocnemius between treatment groups, likely because Alk4 knockdown levels were lower than in the previous study, which used local injection of vivo-morpholinos.

While in our study mice were treated four times with IP over a period of 4 weeks, another study has shown that two subcutaneous doses of siRNA could already be sufficient to lead to silencing in skeletal muscles [15]. Increasing the siRNA dose from 10 to 20 mg/kg did not result in significantly lower levels of *Alk4* in skeletal muscles suggesting that there is a saturation effect on the downregulation that can be achieved with these siRNAs. Additional studies will be required to fully elucidate the most optimal dosing regimen for siRNA delivery to skeletal muscle in dystrophic animals.

In conclusion, our siRNAs were safe and showed significant downregulation of *Alk4* in both nondystrophic and dystrophic muscles, including the diaphragm, which is the most affected muscle in DMD patients, thus making treatment of siRNAs with similar chemical modifications and lipid conjugates for targets involved in the secondary pathology of DMD interesting.

## Supplementary Material

Supplemental data

## References

[1] Birnkrant DJ, K Bushby, CM Bann, SD Apkon, A Blackwell, D Brumbaugh, LE Case, PR Clemens, S Hadjiyannakis, *et al.* (2018). Diagnosis and management of Duchenne muscular dystrophy, part 1: diagnosis, and neuromuscular, rehabilitation, endocrine, and gastrointestinal and nutritional management. Lancet Neurol 17:251–267. 29395989 10.1016/S1474-4422(18)30024-3 PMC5869704

[2] Fortunato F, R Rossi, MS Falzarano and A Ferlini. (2021). Innovative therapeutic approaches for duchenne muscular dystrophy. J Clin Med 10:820. 33671409 10.3390/jcm10040820 PMC7922390

[3] Verhaart IEC and A Aartsma-Rus. (2019). Therapeutic developments for Duchenne muscular dystrophy. Nat Rev Neurol 15:373–386. 31147635 10.1038/s41582-019-0203-3

[4] Duan D. (2018). Systemic AAV micro-dystrophin gene therapy for Duchenne muscular dystrophy. Mol Ther 26:2337–2356. 30093306 10.1016/j.ymthe.2018.07.011 PMC6171037

[5] Mendell JR, Z Sahenk, K Lehman, C Nease, LP Lowes, NF Miller, MA Iammarino, LN Alfano, A Nicholl, *et al.* (2020). Assessment of systemic delivery of rAAVrh74.MHCK7.micro-dystrophin in children with Duchenne muscular dystrophy: a nonrandomized controlled trial. JAMA Neurol 77:1122–1131. 32539076 10.1001/jamaneurol.2020.1484 PMC7296461

[6] McDonald CM, C Campbell, RE Torricelli, RS Finkel, KM Flanigan, N Goemans, P Heydemann, A Kaminska, J Kirschner, *et al.* (2017). Ataluren in patients with nonsense mutation Duchenne muscular dystrophy (ACT DMD): a multicentre, randomised, double-blind, placebo-controlled, phase 3 trial. Lancet 390:1489–1498. 28728956 10.1016/S0140-6736(17)31611-2

[7] Shimizu-Motohashi Y, H Komaki, N Motohashi, S Takeda, T Yokota and Y Aoki. (2019). Restoring dystrophin expression in duchenne muscular dystrophy: current status of therapeutic approaches. J Pers Med 9:1. 30621068 10.3390/jpm9010001 PMC6462907

[8] Spinazzola JM and LM Kunkel. (2016). Pharmacological therapeutics targeting the secondary defects and downstream pathology of Duchenne muscular dystrophy. Expert Opin Orphan Drugs 4:1179–1194. 28670506 10.1080/21678707.2016.1240613 PMC5487007

[9] Tidball JG and M Wehling-Henricks. (2004). Evolving therapeutic strategies for Duchenne muscular dystrophy: targeting downstream events. Pediatr Res 56:831–841. 15531741 10.1203/01.PDR.0000145578.01985.D0

[10] Kole R, AR Krainer and S Altman. (2012). RNA therapeutics: beyond RNA interference and antisense oligonucleotides. Nat Rev Drug Discov 11:125–140. 22262036 10.1038/nrd3625 PMC4743652

[11] Khan T, H Weber, J DiMuzio, A Matter, B Dogdas, T Shah, A Thankappan, J Disa, V Jadhav, *et al.* (2016). Silencing myostatin using cholesterol-conjugated siRNAs induces muscle growth. Mol Ther Nucleic Acids 5:e342. 27483025 10.1038/mtna.2016.55 PMC5023400

[12] Hammond SM, A Aartsma-Rus, S Alves, SE Borgos, RAM Buijsen, RWJ Collin, G Covello, MA Denti, LR Desviat, *et al.* (2021). Delivery of oligonucleotide-based therapeutics: challenges and opportunities. EMBO Mol Med 13:e13243. 33821570 10.15252/emmm.202013243 PMC8033518

[13] Nikam RR and KR Gore. (2018). Journey of siRNA: clinical developments and targeted delivery. Nucleic Acid Ther 28:209–224. 29584585 10.1089/nat.2017.0715

[14] Baltusnikas J, A Fokin, J Winkler and J Liobikas. (2017). Long-term regulation of gene expression in muscle cells by systemically delivered siRNA. J Control Release 256:101–113. 28456678 10.1016/j.jconrel.2017.04.037

[15] Biscans A, J Caiazzi, N McHugh, V Hariharan, M Muhuri and A Khvorova. (2021). Docosanoic acid conjugation to siRNA enables functional and safe delivery to skeletal and cardiac muscles. Mol Ther 29:1382–1394. 33348054 10.1016/j.ymthe.2020.12.023 PMC8058398

[16] Pasteuning-Vuhman S, JW Boertje-van der Meulen, M van Putten, M Overzier, P Ten Dijke, SM Kielbasa, W Arindrarto, R Wolterbeek, KV Lezhnina, *et al.* (2017). New function of the myostatin/activin type I receptor (ALK4) as a mediator of muscle atrophy and muscle regeneration. FASEB J 31:238–255. 27733450 10.1096/fj.201600675R PMC5161514

[17] Elkayam E, R Parmar, CR Brown, JL Willoughby, CS Theile, M Manoharan and L Joshua-Tor. (2016). siRNA carrying an (E)-vinylphosphonate moiety at the 5′ end of the guide strand augments gene silencing by enhanced binding to human Argonaute-2. Nucleic Acids Res 45:3528–3536. 10.1093/nar/gkw1171 PMC5389677 27903888

[18] Soutschek J, A Akinc, B Bramlage, K Charisse, R Constien, M Donoghue, S Elbashir, A Geick, P Hadwiger, *et al.* (2004). Therapeutic silencing of an endogenous gene by systemic administration of modified siRNAs. Nature 432:173–178. 15538359 10.1038/nature03121

[19] Ruijter JM, C Ramakers, WM Hoogaars, Y Karlen, O Bakker, MJ van den Hoff and AF Moorman. (2009). Amplification efficiency: linking baseline and bias in the analysis of quantitative PCR data. Nucleic Acids Res 37:e45. 19237396 10.1093/nar/gkp045 PMC2665230

[20] Kuijper EC, AJ Bergsma, WWMP Pijnappel and A Aartsma-Rus. (2021). Opportunities and challenges for antisense oligonucleotide therapies. J Inherit Metab Dis 44:72–87. 32391605 10.1002/jimd.12251 PMC7891411

[21] Zhang MM, R Bahal, TP Rasmussen, JE Manautou and X-b Zhong. (2021). The growth of siRNA-based therapeutics: updated clinical studies. Biochem Pharmacol 189:114432. 33513339 10.1016/j.bcp.2021.114432 PMC8187268

[22] Akinc A, MA Maier, M Manoharan, K Fitzgerald, M Jayaraman, S Barros, S Ansell, X Du, MJ Hope, *et al.* (2019). The Onpattro story and the clinical translation of nanomedicines containing nucleic acid-based drugs. Nat Nanotechnol 14:1084–1087. 31802031 10.1038/s41565-019-0591-y

[23] Nair JK, JL Willoughby, A Chan, K Charisse, MR Alam, Q Wang, M Hoekstra, P Kandasamy, AV Kel'in, *et al.* (2014). Multivalent N-acetylgalactosamine-conjugated siRNA localizes in hepatocytes and elicits robust RNAi-mediated gene silencing. J Am Chem Soc 136:16958–16961. 25434769 10.1021/ja505986a

[24] Biscans A, A Coles, R Haraszti, D Echeverria, M Hassler, M Osborn and A Khvorova. (2019). Diverse lipid conjugates for functional extra-hepatic siRNA delivery in vivo. Nucleic Acids Res 47:1082–1096. 30544191 10.1093/nar/gky1239 PMC6379722

[25] Osborn MF and A Khvorova. (2018). Improving siRNA delivery in vivo through lipid conjugation. Nucleic Acid Ther 28:128–136. 29746209 10.1089/nat.2018.0725 PMC5994667

[26] Golzio M, L Mazzolini, P Moller, MP Rols and J Teissié. (2005). Inhibition of gene expression in mice muscle by in vivo electrically mediated siRNA delivery. Gene Ther 12:246–251. 15592423 10.1038/sj.gt.3302405

[27] Kishida T, H Asada, S Gojo, S Ohashi, M Shin-Ya, K Yasutomi, R Terauchi, KA Takahashi, T Kubo, J Imanishi and O Mazda. (2004). Sequence-specific gene silencing in murine muscle induced by electroporation-mediated transfer of short interfering RNA. J Gene Med 6:105–110. 14716682 10.1002/jgm.456

[28] Hagstrom JE, J Hegge, G Zhang, M Noble, V Budker, DL Lewis, H Herweijer and JA Wolff. (2004). A facile nonviral method for delivering genes and siRNAs to skeletal muscle of mammalian limbs. Mol Ther 10:386–398. 15294185 10.1016/j.ymthe.2004.05.004

[29] Kawakami E, N Kawai, N Kinouchi, H Mori, Y Ohsawa, N Ishimaru, Y Sunada, S Noji and E Tanaka. (2013). Local applications of myostatin-siRNA with atelocollagen increase skeletal muscle mass and recovery of muscle function. PLoS One 8:e64719. 23717655 10.1371/journal.pone.0064719 PMC3661523

[30] Heemskerk H, C de Winter, P van Kuik, N Heuvelmans, P Sabatelli, P Rimessi, P Braghetta, GJ van Ommen, S de Kimpe, *et al.* (2010). Preclinical PK and PD studies on 2'-O-methyl-phosphorothioate RNA antisense oligonucleotides in the mdx mouse model. Mol Ther 18:1210–1217. 20407428 10.1038/mt.2010.72 PMC2889733

