# Supplementary material for: Efficient Downregulation of Alk4 in Skeletal Muscle After Systemic Treatment with Conjugated siRNAs in a Mouse Model for Duchenne Muscular Dystrophy
Source: Nucleic Acid Ther. 2023 Feb 1;33(1):26–34. doi: 10.1089/nat.2022.0021 (PMC9940804; doi:10.1089/nat.2022.0021)
Supplement: Supplemental data [file Supp_FigS1-S3.pptx]

## Slide 1
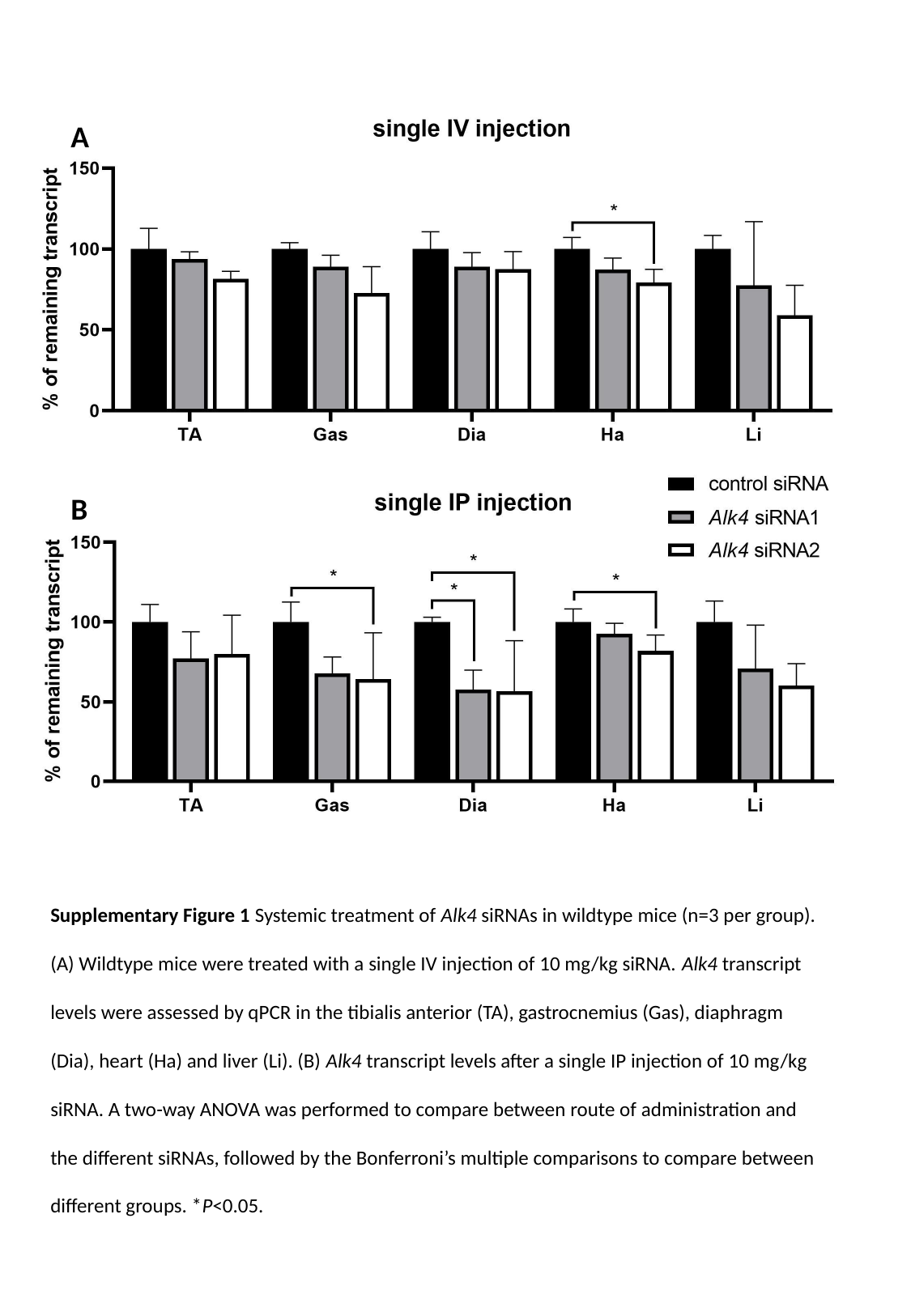

A
A
B
B
Supplementary Figure 1 Systemic treatment of Alk4 siRNAs in wildtype mice (n=3 per group). (A) Wildtype mice were treated with a single IV injection of 10 mg/kg siRNA. Alk4 transcript levels were assessed by qPCR in the tibialis anterior (TA), gastrocnemius (Gas), diaphragm (Dia), heart (Ha) and liver (Li). (B) Alk4 transcript levels after a single IP injection of 10 mg/kg siRNA. A two-way ANOVA was performed to compare between route of administration and the different siRNAs, followed by the Bonferroni’s multiple comparisons to compare between different groups. *P<0.05.

## Slide 2
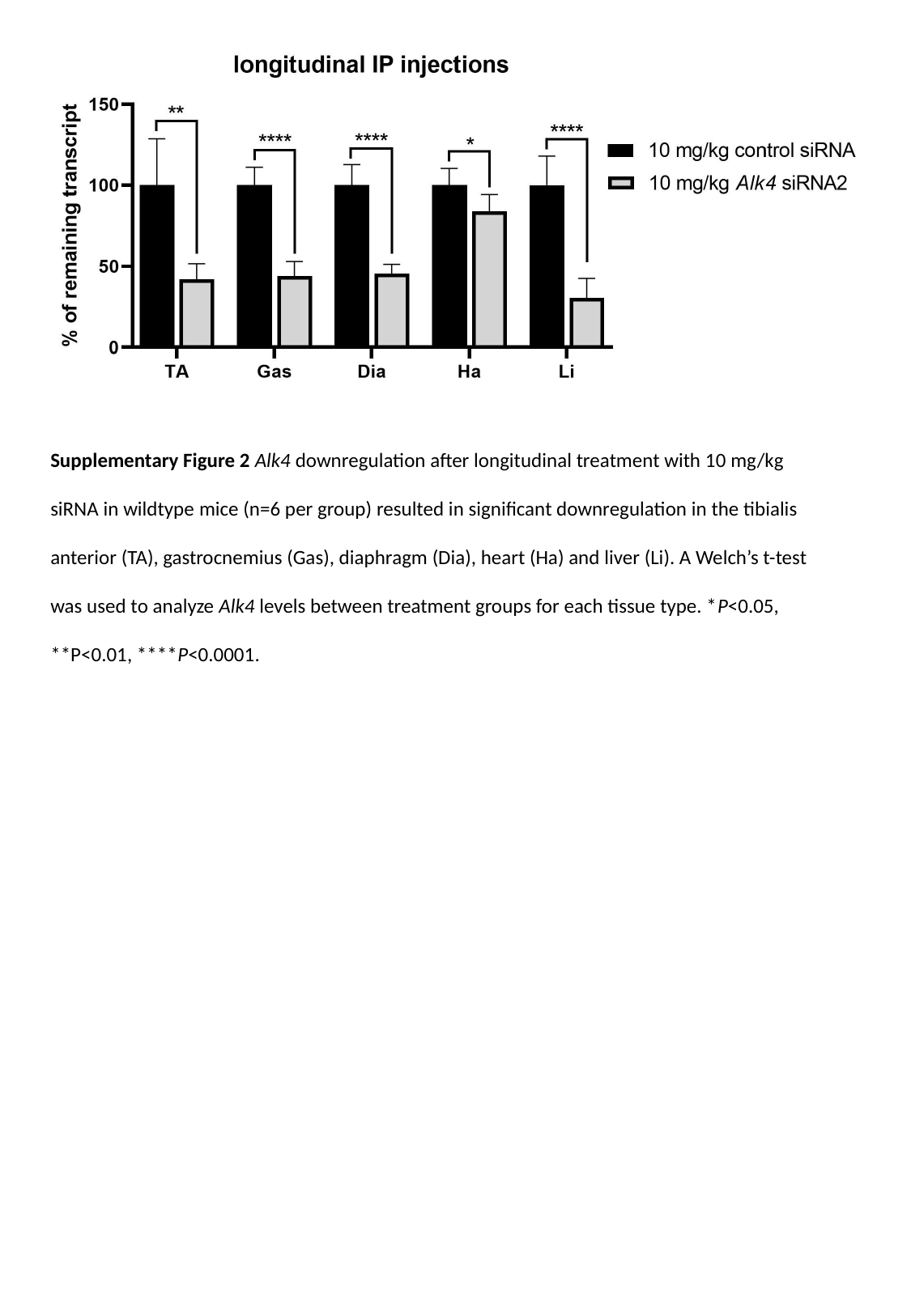

Supplementary Figure 2 Alk4 downregulation after longitudinal treatment with 10 mg/kg siRNA in wildtype mice (n=6 per group) resulted in significant downregulation in the tibialis anterior (TA), gastrocnemius (Gas), diaphragm (Dia), heart (Ha) and liver (Li). A Welch’s t-test was used to analyze Alk4 levels between treatment groups for each tissue type. *P<0.05, **P<0.01, ****P<0.0001.

## Slide 3
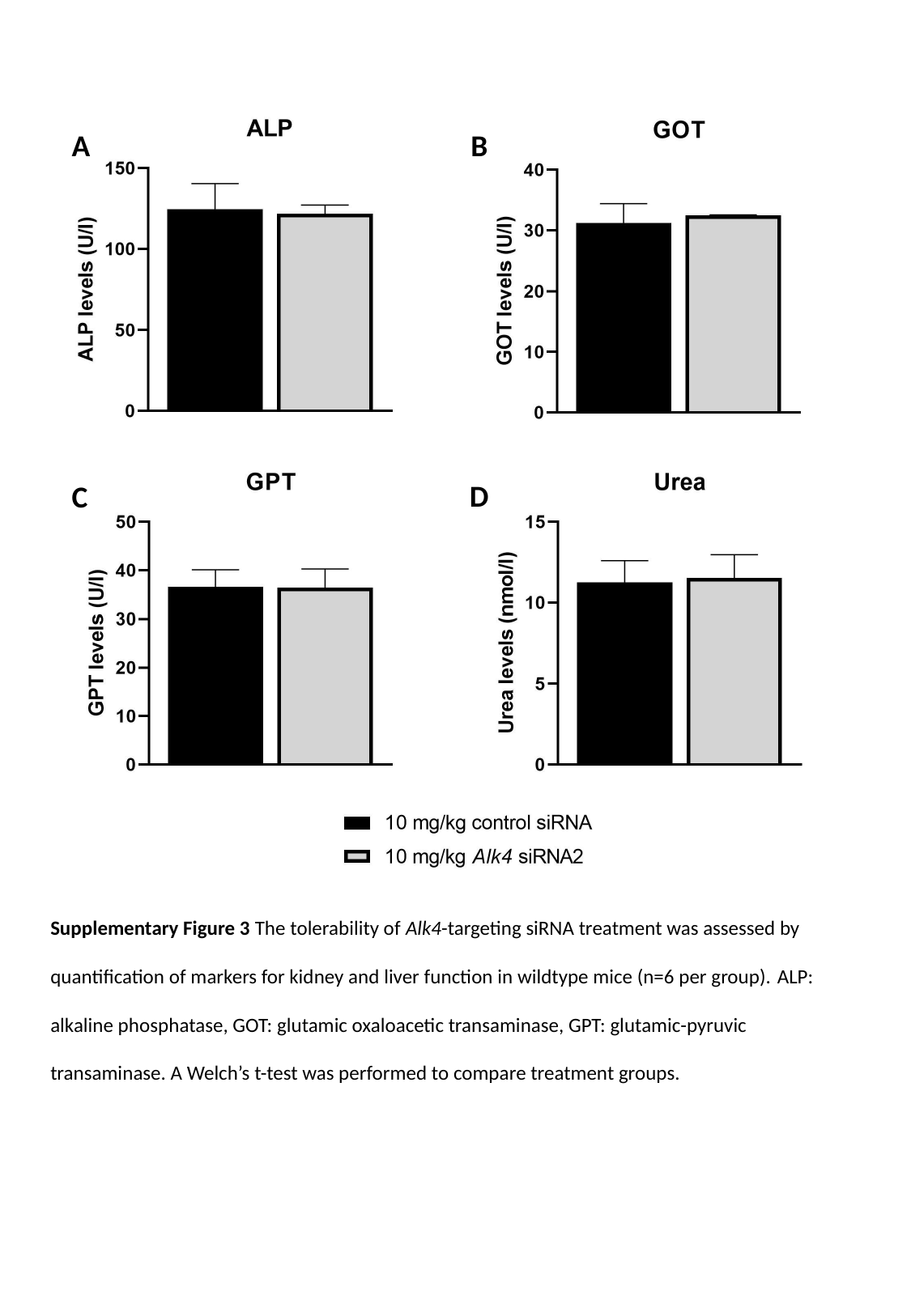

B
A
D
C
Supplementary Figure 3 The tolerability of Alk4-targeting siRNA treatment was assessed by quantification of markers for kidney and liver function in wildtype mice (n=6 per group). ALP: alkaline phosphatase, GOT: glutamic oxaloacetic transaminase, GPT: glutamic-pyruvic transaminase. A Welch’s t-test was performed to compare treatment groups.
